# The multiple roles of the NlpC_P60 peptidase family in mycobacteria – an underexplored target for antimicrobial drug discovery

**DOI:** 10.1002/1873-3468.70021

**Published:** 2025-03-03

**Authors:** Catharina dos Santos Silva, Marcio Vinicius Bertacine Dias

**Affiliations:** ^1^ Department of Microbiology, Institute of Biological Sciences University of São Paulo Brazil

**Keywords:** cell division, cysteine protease, *Mycobacterium tuberculosis*, pathogenesis, peptidoglycan

## Abstract

The main function of the cell wall is to maintain cellular integrity throughout the cell cycle by keeping the cell shape during growth and division. However, far from being a static structure, the cell wall undergoes constant recycling and even molecular modifications of its components. The major component of the bacterial cell wall is the peptidoglycan layer. The balance between peptidoglycan synthesis and degradation is crucial for cell viability and proliferation. Hence, factors involved in the control of peptidoglycan turnover are considered interesting targets for drug development. Members of the NlpC_P60 superfamily of peptidases have been described to participate in the physiology and pathogenesis of several bacterial lineages. However, the knowledge about NlpC_P60‐like proteins from mycobacteria is still limited, despite the great progress in recent years. In this Review, we aimed to compile the information about mycobacterial NlpC_P60 proteins, pointing out their distribution across pathogenic and environmental *Mycobacterium* species, highlighting the knowledge gaps and describing their structural features, role in the physiology and mycobacterial pathogenesis.

## Graphical abstract

The NlpC_P60 superfamily of peptidases is recognised by its key role in bacterial cell wall homeostasis. Recently, studies have also described the involvement of NlpC_P60‐like enzymes in bacterial competitive mechanisms and pathogenesis across several lineages. In our Review, we provided compiled information about the mycobacterial NlpC_P60 proteins, shedding light on the underexplored potential of these peptidases as targets for drug design campaigns.

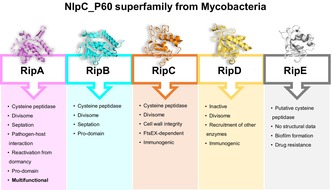

## Abbreviations

**AEC**, alveolar epithelial cells

**Cglu**, *Corynebacterium glutamicum*

**CHAP**, cysteine, histidine‐dependent amidases/peptidases

**Cryo‐EM**, cryogenic electron microscopy

**eCIS**, extracellular contractile injection system

**FN3**, fibronectin type III

**GSPS**, glutathionylspermidine synthases

**HMWPG**, high molecular weight peptidoglycan

**LRAT**, lecithin retinol acyltransferase

**MABC**, *Mycobacterium abscessus* complex

**MAC**, *Mycobacterium avium* complex

**MTB**, *Mycobacterium tuberculosis*

**NAG**, *N*‐acetyl glucosamine

**NAM**, *N*‐acetyl/glycolyl muramic acid

**NTM**, nontuberculous mycobacterial

**PBP**, penicillin‐binding protein

**Rip**, Rpf‐interacting protein

**Rpf**, resuscitation‐promoting factor

**STPK**, serine–threonine protein kinase

**T6SS**, type VI secretion system

**TAT**, twin‐arginine translocation

**TB**, tuberculosis

**TGM**, transglutaminase

**TLR4**, toll‐like receptor 4

**vWA**, Von Willebrand factor type A

**XRE**, xenobiotic response element

Most members of the genus *Mycobacterium* are nonpathogenic bacteria species, usually environmental free‐living microorganisms [1]. However, there are a few known obligate pathogenic mycobacteria that can cause infections in humans and animals with high morbidity and mortality worldwide [2]. This is the case of *Mycobacterium tuberculosis* (MTB) that causes tuberculosis (TB), the world's deadliest bacterial infection, which usually affects the lungs but is also able to affect other human organs and tissues. Although this disease is caused predominantly by MTB, other mycobacteria such as *M. bovis* and *M. avium*, which primarily infect cattle and birds, respectively, can also be able to cause similar diseases [3]. Leprosy is another clinically relevant infectious disease caused by mycobacteria, particularly *M. leprae* and *M. lepromatosis*. This disease causes severe neurological and dermatological damage, including loss of sensitivity to heat or pain and tissue deformities as a consequence of the invasion of Schwan cells and histiocytes by *M. leprae* [4, 5, 6]. Overall, the understanding of the *Mycobacterium* genus has focused on studies of clinically relevant species. However, despite bringing together centuries of research on the biology of pathogenic mycobacteria, the recent global increase in the incidence and prevalence of nontuberculous mycobacterial (NTM) infections have also raised concerns about the emergence of other potential pathogenic species [7]. Although most NTMs are ubiquitous, certain species are nonhuman pathogens, such as *M. marinum*, while others are opportunistic in animals and humans [8, 9]. NTM infections have a wide clinical manifestation, but the most common symptom is lung disease often caused by species from the *M. avium* complex (MAC), *M. abscessus* complex (MABC), *M. kansasii*, *M. xenopi* and *M. malmoense* [7, 8, 9, 10, 11]. Thus, the rise of environmental species of mycobacteria in the clinical scenario has awakened the need to build a broader knowledge of the group [8, 12].

**Fig. 1:**
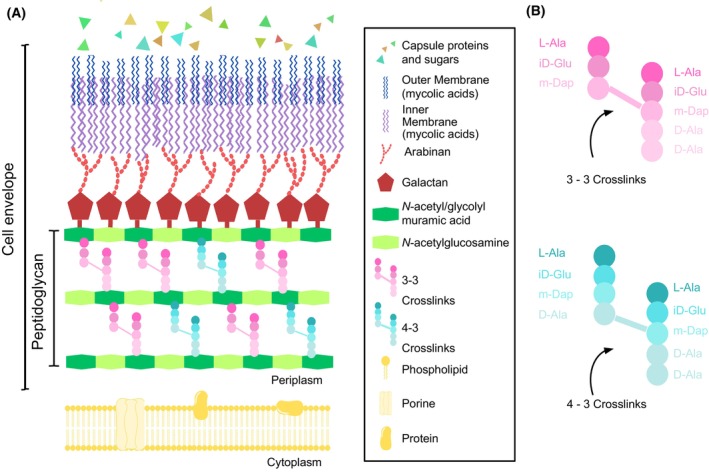
Mycobacterial cell envelope. (A) Schematic representation of the different layers constituting the mycobacterial cell envelope. Above the cell membrane (CM) is found the peptidoglycan (PG) layer, a polymer composed of *N*‐acetyl‐glucosamine disaccharide and a mixture of *N*‐glycolyl/acetyl‐muramic acid disaccharides. Surrounding peptidoglycan is located in the polysaccharide arabinogalactan (AG), formed by galactan and arabinan sugars. A mycomembrane (MM) of long‐chain fatty acids encompasses the cell wall core and can be further surrounded by a capsule (CA) of polysaccharides and proteins in pathogenic species. (B) Composition of the stem peptides cross‐linking peptidoglycan in mycobacteria.

Mycobacteria are recognised by their particular and complex cell envelope architecture (Fig. 1). The peptidoglycan layer is formed by long polymers of repeating *N‐*acetyl glucosamine (NAG) and *N*‐acetyl/glycolyl muramic acid (NAM) units connected by β‐1,4 glycoside linkages and heavily cross‐linked with peptide stems composed of L‐alanyl‐γ‐D‐isoglutamyl‐*meso*‐diaminopimelyl‐D‐alanine [13, 14]. Interestingly, mycobacterial peptidoglycan side chains are more abundant in noncanonical 3–3 crosslinks than traditional 4–3 crosslinks [13, 15, 16] (Fig. 1B). Baranowski *et al*. [17] showed that 4–3 crosslinks are predominantly created at polar growth sites by penicillin‐binding proteins (PBPs) and then are cleaved by D, D‐endopeptidases along with cell wall ageing, becoming substrates for L, D‐transpeptidases to produce 3–3 crosslinks at older peptidoglycan. A further layer of arabinogalactan is anchored to the peptidoglycan and this structure is constituted by galactan chains of repeating 5‐ and 6‐D‐galactopyranosyl disaccharides and branched arabinan covalently linked to an outer membrane of long‐carbon‐mycolic acids. This ‘mycomembrane’ is responsible for the extremely lipidic nature of the mycobacterial cell wall core. Moreover, the protein and polysaccharide capsule‐like structure can also be found enclosing the cell wall of pathogenic species [14, 18].

Components of the cell wall undergo constant remodelling during bacterial growth and division. Therefore, the balance between peptidoglycan hydrolysis and synthesis is crucial for maintaining cell shape and survival. Mycobacterial cells elongate in the poles and display an asymmetric manner for daughter‐cell separation, unlike most rod‐shaped bacteria [18]. A large number of proteins and enzymes play together in order to control these processes. However, understanding the cell cycle dynamics through the coordination of these macromolecules remains elusive, especially in mycobacteria.

**Fig. 2:**
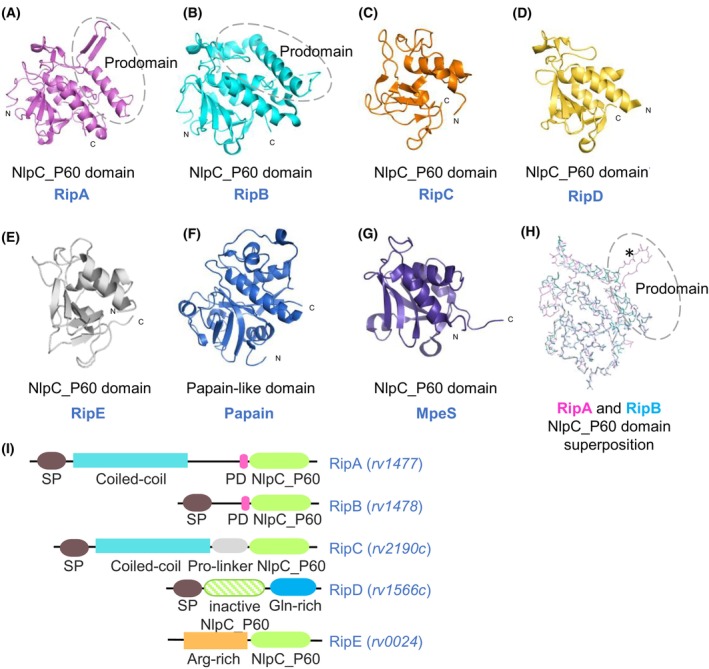
Structure comparison of the NlpC_P60 proteins from *M. tuberculosis* and related proteinases. (A) Crystal structure of the catalytic domain of *M. tuberculosis* RipA (PDB: 3NE0) with an additional regulatory region [32]. (B) Crystal structure of *M. tuberculosis* RipB (PDB: 3PBI) with an additional regulatory region [33]. (C) The catalytic domain of *M. tuberculosis* RipC from the Cryo‐EM structure of FtsEX‐RipC complex (PDB: 8IDC) [34]. (D) Crystal structure of *M. tuberculosis* RipD (PDB: 4JXB) [35]. (E) Structure prediction by AlphaFold [36] for *M. tuberculosis* RipE (accession code P71594) (pTM = 0.59). (F) Crystal structure of papain‐like protease (PDB: 1PPN) [37]. (G) NMR structure of *E. coli* MpeS (Spr) (PDB: 2K1G) [38]. (H) Superposition of RipA (magenta) and RipB (cyan) main chains highlighting that their blocking loops have differences in structure as the β‐hairpin present in the RipA loop is absent in RipB (marked by an asterisk). Dashed circle around the prodomain of RipA and RipB in A, B and H. (I) Schematic representation of domain organisation of the MTB NlpC_P60 proteins. The abbreviations are: Arg, arginine; Gln, glutamine; PD, prodomain; Pro, proline; SP, signal peptide.

The NlpC_P60 (New lipoprotein C from *Escherichia coli*/Protein of 60‐kDa from *Listeria monocytogenes*) superfamily consists of cell wall hydrolases that do not only play a key role in the mycobacterial cell wall homeostasis but also in the bacterial physiology [19, 20, 21, 22, 23, 24], competitive mechanisms [24, 25, 26] and pathogenesis of numerous bacterial lineages [20, 27, 28, 29, 30]. These peptidases share a Papain‐like cysteine protease domain in which an N‐terminal region constituted by α‐helices is followed by a 4‐ to 6‐stranded antiparallel β‐sheet arranged as a barrel‐like scaffold [20, 31] (Fig. 2). The catalytic core of NlpC_P60 domains has the canonical Cys‐His dyad similar to those described in cysteine peptidases, where the cysteine residue acts as a nucleophile to cleave the peptidoglycan stem peptides whereas histidine plays a role as a base [20, 31].

Despite high conservation in the structural core and mechanism of catalysis, the NlpC_P60 domains display differences in substrate preference, encompassing a large set of proteins of distinct but related catalytic activities, such as murein hydrolases, acyltransferases, amidases, transglutaminases and ubiquitinases [19, 20, 31, 39]. This can be related to the occurrence of circularly permuted topologies (where the catalytic cysteine and histidine positions are swapped) and lineage‐specific divergence of domain architectures due to variations in the cell wall composition and the existence of additional molecular modifications in the peptidoglycan components across bacterial lineages [39]. In addition, NlpC_P60 domains can be found in larger proteins associated with regulatory regions or domains, which can also be subject to post‐translational processing as an additional layer of regulation of their activity (Fig. 2I) [31, 39]. Taken together, the broad biochemical diversity of the superfamily members indicates that multiple NlpC_P60‐like proteins perform nonoverlapping roles in the bacterial cell.

In mycobacteria, Gao et al. first described NlpC_P60 enzymes in *M. marinum*. They found the bicistronic operon *iipA* and *iipB* (invasion and intracellular persistence), which is involved with invasion and persistence in macrophages [27]. An orthologue bicistronic operon encoded by *ripA* (*rv1477*) and *ripB* (*rv1478*) was also found in MTB [27]. Indeed, five genes of apparently nonredundant functions were identified as members of the NlpC_P60 family in the MTB genome [40]. So far, the one that encodes the protein RipA is the most studied mycobacterial NlpC_P60‐domain‐containing enzyme. RipA is considered a multifunctional protein as it was predicted to have a role in macrophage invasion and persistence, mycobacterial cell reactivation from dormancy and daughter‐cell separation during vegetative growth [18, 20, 21, 27, 30, 40, 41]. Because of its crucial importance in several processes, there is a heightened interest in the study of this enzyme as a target for drug discovery, along with the understanding of the molecular basis of tuberculosis pathogenesis.

In this Review, we provide an overview of the peptidoglycan‐related NlpC_P60 family members from mycobacteria. For that, we discuss the conservation of these peptidase orthologues among different *Mycobacterium* species and describe the structural and functional features of the best‐known NlpC_P60‐like proteins, which are those from MTB. Furthermore, this paper highlighted the potential of NlpC_P60 peptidases as targets for drug design campaigns against both long‐established and emerging mycobacterial pathogenic species.

## Distribution of the NlpC_P60‐like endopeptidases across mycobacteria and related organisms

The NlpC_P60 superfamily is a highly divergent and ubiquitous group of papain‐like cysteine peptidases. Early phylogenetic analysis of this superfamily by Anantharaman and Aravind identified four major families: p60‐like, AcmB/LytN‐like, GSPSs amidase‐like and lecithin retinol acyltransferase‐like (LRAT‐like) [39]. Among these, members of the p60‐like family are the most prevalent within bacterial lineages. Notably, mycobacterial NlpC_P60‐like proteins belong to the p60 family and are part of a patchy phyletic group (SCP1.148), which includes other actinobacteria, such as *Streptomyces coelicolor*, and the Gram‐positive bacterium *Clostridium acetobutylicum* [39].

Although the NlpC_P60‐domain‐containing proteins remain underexplored in mycobacteria, the most studied examples are from the clinically significant pathogen MTB. As previously mentioned, the MTB genome encodes at least five NlpC_P60 endopeptidases: Rv1477 (*ripA*), Rv1478 (*ripB*), Rv2190c (*ripC*), Rv1566c (*ripD*) and Rv0024 (here referred to as *ripE*) [27, 28, 40, 42, 43, 44] (Fig. 2). The acronym ‘Rip’ originates from the ability of RipA to interact with resuscitation‐promoting factors (Rpf), hence referred to as Rpf‐interacting Proteins [21, 41]. Notably, all five genes encoding NlpC_P60‐like proteins are retained in pathogenic mycobacteria, except *M. abscessus* and *M. leprae* [44]. In contrast, environmental species exhibit extensive multiplicity for *ripA ‐ D* genes but apparently lack orthologues of *ripE* [44].

Further phylogenetic analysis of NlpC_P60‐like enzymes from the *Corynebacteriales* order revealed high gene redundancy among bacterial families [45]. For instance, genomes of *Corynebacteriaceae* typically encode four genes, most *Mycobacteriaceae* contain five and *Nocardiaceae* genomes may encode more than eight [45]. The major clade includes RipA orthologues, such as *Clostridium diphtheriae* DIP1281 and *Corynebacterium glutamicum* (Cglu) Cg1735. Interestingly, a duplication event resulted in the formation of the *ripAB* bicistronic operon, which is syntenic exclusively among *Mycobacteriaceae* genomes, explaining the absence of RipB in other *Corynebacteriales* families [45].

Altogether, these data support the broad role of NlpC‐like enzymes in mycobacterial physiology. As previously mentioned, these enzymes act in a variety of pathways, including mediators of normal cell growth, cell division, virulence factors and pathogenesis in clinically relevant and emergent species. In addition, the presence or absence of specific *rip* genes reflects the essentiality of their products and, therefore, corroborates the nonoverlapping function of the Rip family.

## Deciphering the biology of the NlpC_P60 proteins from *Mycobacterium tuberculosis*

### The highly conserved bicistronic operon of nonredundant rip proteins—RipA And RipB

The *ripAB* locus is highly conserved across mycobacteria [27, 44, 45]. The maintenance of a bicistronic operon encoding for similar peptidoglycan peptidases strongly suggests the nonredundant role of RipA and RipB and the importance of both enzymes for bacterial viability [44, 45]. Early studies have proposed *ripA*, but not *ripB*, as essential genes for *M. smegmatis* and MTB [21, 44]. Depletion of *ripA* orthologue in *M. smegmatis* impaired vegetative growth while resulting in an elongated multiseptated phenotype and increased antibiotic susceptibility in MTB [21, 30]. Eventually, genetic studies have demonstrated that *ripA* and *ripB* genes are individually nonessential in *M. smegmatis*, whereas loss of both gene products is lethal [46]. Interestingly, the strains with individual *ripA* and *ripB* knockouts display distinct phenotypes [30], which reinforce the nonredundant functionality roles of the Rip enzymes. Although RipA and RipB have some overlapping functions in viability [27, 30, 46], they should differ, at least in their role in persistence.

**Fig. 3:**
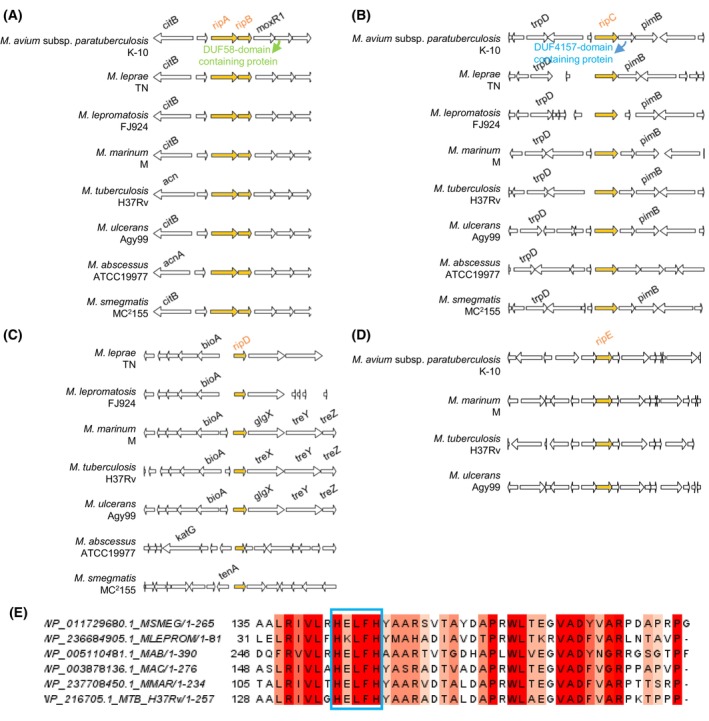
Genomic context of the NlpC_P60‐coding genes in the genomes of environmental and pathogenic mycobacteria. The genomes chosen for the analysis were selected based on those containing NlpC_P60 coding genes in the COG (Cluster of Orthologous Genes) Database search (COG0791) [47, 48]. The genomic context was performed using the genespy Software (https://lbbe‐dmz.univ‐lyon1.fr/GeneSpy/about.html) [49] for the strains of *M. avium* subsp. *paratuberculosis* K‐10 (WP_003876096.1/NC_002944.2), *M. leprae* TN (WP_010908553.1/NC_002677.1), *M. lepromatosis* FJ924 (WP_045843411.1/NZ_CP083405.1), *M. marinum* M (WP_041324584.1/NC_010612.1), *M. tuberculosis* H37Rv (NP_215994.1/NC_000962.3), *M. ulcerans* Agy99 (WP_011739629.1/NC_008611.1), *M. abscessus* ATCC19977 (WP_005093513.1/NC_010397.1) and *M. smegmatis* MC^2^155 (WP_011728846.1/NC_008596.1). Genes encoding for the query NlpC_P60‐coding proteins are shown in orange. The accession numbers of the anchor genes in each neighbourhood shown were NP_215994.1 (MTB *ripB*, for *ripAB* genomic context), NP_216706.1 (MTB *ripC*, for *ripC* genomic context), NP_216082.1 (MTB *ripD*, for *ripD* genomic context) and NP_214538.1 (MTB *rv0024*, for *ripE* genomic context). (A) *ripA* and *ripB* genes are located in a highly conserved bicistronic operon found in a preserved gene neighbourhood in all the considered genome sequences, where the target operon is regularly upstream of the *moxR* */moxR1* gene, which is shown to be related to RipA processing. (B) The genome vicinity of *ripC* is also very conserved among mycobacteria. Apart from *M. leprae*, *ripC* is often close to a hypothetical protein followed by mannosyltransferase (*pimB*)/glycosyltransferase coding genes. (C) The genomic context of *ripD* is only conserved among pathogenic *Mycobacterium* genomes, where it is located in between biotin and trehalose biosynthesis gene clusters. Note the genomic context of *ripD* from *M. avium* subsp. *paratuberculosis* K‐10 (MAP_1272c/MAP_RS06455) could not be generated by the software. (D) *ripE* is only found in pathogenic genome species, except *M. lepra*e and *M. lepromatosis*. The gene neighbourhood of *ripE* is not conserved. In MTB, *ripE* genomic context includes genes encoding for proteins related to peptidoglycan synthesis. (E) The amino acid sequences of hypothetical proteins encoded by genes downstream of *ripC* from *M. smegmatis* (MSMEG), *M. lepromatosis* (MLEPROM), *M. abscessus* (MAB), *M. avium complex* (MAC), *M. marinum* (MMAR) and *M. tuberculosis* (MTB H37Rv) genomes were aligned by using clustal omega (EMBL‐EBI, Cambridgeshire, UK). The degree of conservation is shown in coral to red. The zinc‐binding HExxH motif of the DUF4157‐domain‐containing proteins found in the analysed sequences is highlighted in the blue box. The figure was prepared by jalview (https://www.jalview.org/).

Moreover, the *ripAB* loci share the same gene neighbourhood (Fig. 3A) among mycobacterial species. For instance, the *moxR1/moxR* operon is regularly downstream of *ripAB* in all the considered genomes. Besides its multiple cellular functions, MoxR1 ATP‐dependent chaperone has been reported to interact with RipA and mediates its transport through the twin‐arginine translocation (TAT) secretion system [50]. This further suggests a similar post‐translational mechanism for RipA activity in other mycobacteria. Curiously, despite sharing the same pathway for RipA transport, it was observed that full‐length _MTB_RipA overexpression in *M. smegmatis* does not cause toxicity or host cell phenotype alteration. The hypothesis is that the regulatory factors in *M. smegmatis* could not be able to process and further activate its orthologue in the periplasm. Based on that, it is suggested that highly specific protein–protein interactions evolved to control RipA hydrolase activity between the different species [51].

In addition, the gene organisation in the *moxR* operon resembles that of the species‐specific PFNA gene cluster found in bifidobacteria. The recently described *PFNA* operon has five core genes, which encode for serine–threonine protein kinase (STPK) Pkb2, fibronectin type III (FN3), AAA‐ATPase, DUF58‐containing protein and a transglutaminase (TGM) [52, 53]. Studies have shown that the PFNA gene cluster is involved in the recognition and modulation of host immune signals [52, 53, 54]. Interestingly, AAA‐ATPase in the PFNA gene cluster belongs to the MoxR subfamily and is recognised to be the substrate for Pkb2 [53]. In mycobacteria, the *moxR1* gene is also adjacent to a sequence encoding for a DUF58‐containing protein (Fig. 3A). However, to date, no evidence has clarified the functional relationship between these two proteins or the specific role of the DUF58‐containing protein.

Although the DUF58 domain (IPR002881) remains functionally unidentified, it appears to be associated with hypothetical proteins containing the Von Willebrand factor type A (vWA) domain, as documented in the INTERPRO database. Supporting this, the gene located downstream of the DUF58‐containing protein in mycobacterial *moxR* operon encodes a hypothetical membrane protein that also harbours a vWA domain (IPR002035). The vWA domain is recognised as a MoxR partner and functions as a cochaperone, facilitating protein–protein interactions through metal binding [55, 56].

In *Escherichia coli*, the genes corresponding to these two proteins (RavA/MoxR and ViaA/vWA) are organised within the same operon (*ravAviaA*) and play roles in acidic stress response and respiratory processes [56]. On the other hand, according to the phylogenetic analysis of the MoxR AAA+ chaperone family performed by Snider and Houry (2006), the gene organisation of the *moxR* operon in mycobacteria suggests it belongs to the MRP subfamily due to the additional presence of the DUF58 encoding gene [55].

Furthermore, the MoxR‐vWA complex has recently been associated with biological conflict mechanisms. The locus in question includes three core components, suggesting it could form a ternary MoxR‐vWA subsystem widely present in Actinobacteria [57]. Although the mycobacterial operon arrangements are not entirely consistent with the genomic contexts reported previously, evidence supporting the hypothesis that MoxR participates in thresholding mechanisms related to biological conflict includes the fact that _MTB_MoxR1 inhibits TLR4‐mediated autophagy in macrophages, interfering with the production of pro‐inflammatory cytokines [58]. Interestingly, it has also been proposed that RipA elicits the production of protective cytokines through TLR4 activation [59]. Regardless of the known interaction between RipA and MoxR1 and their role as host immune modulators in MTB infections, further investigation is required to determine whether these two multifunctional proteins and their related entire gene cluster would belong to an evolved pathway to handle the host responses by conflict‐related mechanisms or by chaperone‐mediated activation of RipA. In addition, such data will also contribute to building a broader understanding of the evolutionary history and function of the *ripAB* locus.

Both RipA and RipB are secreted and cell‐associated peptidoglycan hydrolases required for daughter‐cell separation in mycobacteria [18, 21, 27, 30, 40]. RipA is found at the poles of growing cells as well as co‐localises with RpfB at the dividing septum [18, 21]; therefore, the endopeptidase is also reported to participate in mycobacteria reactivation from dormancy [18]. In fact, RipA was early described as a binding partner for the peptidoglycan glycosidase RpfB (Rpf‐interacting protein A—RipA) [21, 41] but has emerged from a supporting role in the control of cell division to become a protagonist and one of the most intriguing mycobacterial enzymes. Further, the synergistic action of the RipA:RpfB complex—in favour of cell division—is inhibited by the interaction between RipA with the antagonist protein, PonA1 [41, 60]. PonA1 is a high molecular weight penicillin‐binding protein (PBP), which means it has transpeptidase and transglycosylase enzymatic domains [61]. Coprecipitation results and enzymatic assays indicated that either the carboxy‐terminal region of the PonA1 transpeptidase domain or the catalytic domain of RpfB interacts with residues of the endopeptidase domain (catalytic domain) of RipA [60]. However, there is still no structural data on these protein–protein complexes, which limits the understanding of how they are formed and regulated.

While the partners of RipA have been thoroughly studied to elucidate its endopeptidase regulation and function, much less is known about the localization and potential partners, if any, of RipB. Consequently, whether RipB interacts with the RipA partners or can replace it in function remains unclear.

The overall architecture of RipA consists of a signal peptide, a noncatalytic coiled‐coil amino‐terminal domain, and a carboxy‐terminal region containing a prodomain and a catalytic NlpC_P60‐like domain [27, 62, 63, 64] (Fig. 2I). Nevertheless, RipB comprises a signal peptide and a catalytic NlpC_P60 domain (Fig. 2I). Unlike RipA, the coiled‐coil amino‐terminal region is absent in RipB [27, 40, 63].

**Fig. 4:**
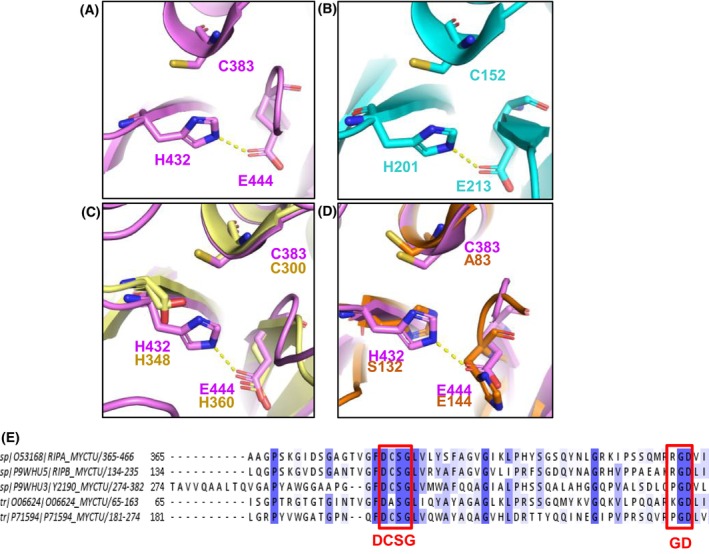
Comparison of catalytic residues of the Rip enzymes from *M. tuberculosis*. (A, B) Zoomed catalytic site clefts of RipA (magenta) and RipB (cyan) showing the conserved catalytic triad Cys‐His‐Glu residues in ball‐and‐sticks. (C) Superposition of the catalytic residues of RipA and RipC, which carries the canonical NlpC_p60 triad Cys‐His‐His. (D) Superposition of the catalytic residues of RipA and RipD, revealing loss of RipD peptidase activity upon modification of triad residues. (E) Multiple sequence alignment (jalview) of MTB Rip proteins generated by clustal omega (embl‐ebi): RipA (top sequence), RipB, RipC (Y2190), RipD (O06624) and RipE (P71594). The degree of conservation is shown in sheds of blue. Motifs DCSG and RGD are highlighted by red boxes.

The core structure of the NlpC_P60 catalytic domain of both RipA and RipB has an α + β organisation composed of three α‐helices surrounding a central 6‐strained antiparallel β‐sheet core (Fig. 2) [40, 62, 63]. As expected, the motif Asp‐Cys‐Ser‐Gly (DCSG) required for NlpC_P60 peptidoglycanase activity is located at the N‐terminal of the helix α2 [27, 62, 63] (Fig. 4A). However, the enzymes also require a tripeptide Arg‐Gly‐Asp (RGD) motif for its proper function (Fig. 4A) [27, 62, 63]. The RGD motif is well‐known to mediate cell adhesion through integrin receptor recognition at the host cell surface [65, 66], enabling pathogen uptake [67]. Previous studies have shown that mutations in the RGD and DCSG motifs of IipA, a RipA orthologue from *M. marinum*, lead to abnormal cell shape and prevent this bacterium from invading the macrophage, suggesting that these two motifs are essential for both peptidase activity and invasion of host cells [27].

Although RipB shares about 70% identity with the catalytic domain of RipA, there are significant structural dissimilarities between them [27, 63] (Fig. 2H). For instance, the prodomain region is recognised to be the inactivation loop of the RipA core domain and consists of a β‐hairpin and a long amphipathic α‐helix (Fig. 2A) [40, 62]. Likewise, this segment wraps around the NlpC_P60 core domain in RipB but differs in structure since it forms two α‐helices (Fig. 2B) [40, 63]. These features of both enzymes lead to important differences between their cavities, which provide a distinct substrate‐binding environment for each one [63].

In spite of having an NlpC_P60‐like catalytic domain, RipA catalytic triad (Cys^383^‐His^432^‐Glu^444^) resembles that of CHAP domain‐containing proteins (cysteine, histidine‐dependent amidases/peptidases, Cys‐His‐Asp catalytic triad) (Fig. 4) [62, 63, 68]. These proteins are an alternative class of autolysins in which activity occurs upon a mechanism of cysteine nucleophilic attack [68]. Indeed, the known crystallographic structure of RipA suggests the formation of a thiolate as the nucleophile upon polarisation of the thiol group of the catalytic cysteine by the imidazole of the catalytic histidine [62]. Surprisingly, the third catalytic residue (Glu^444^) is also crucial for the enzyme activity. As shown by Squeglia et al. [69], Glu^444^ plays a key role in orienting the catalytic cysteine in a D‐configuration by restraining the catalytic site cleft flexibility through an ionic interaction with an active‐site arginine. Further analysis of mutants showed that the L‐to‐D inversion of cysteine leads to the loss of enzymatic activity [69]. Thus, consistent with the D‐γ‐glutamyl‐containing peptides CHAP and NlpC_P60 enzymes substrates [68], RipA works as a D, L‐endopeptidase which cleavages at the bond between D‐glutamate and D‐diaminopimelate residues from the peptidoglycan stem peptide [63, 69, 70]. Similarly, RipB also contains the Cys^152^‐His^201^‐Glu^213^ catalytic triad [27, 63] (Fig. 4). Based on that, it is not surprising that RipB is also capable of binding to the peptidoglycan and cleaving the bond between D‐glutamate and m‐diaminopimelic residues, hence acting as a D, L‐endopeptidase [47, 68]. Accordingly, enzymatic assays showed both enzymes are enabled to hydrolyse peptidoglycan fragments [63]. Nevertheless, only RipA demonstrated the ability to cleave high molecular weight peptidoglycan (HMWPG) from *Bacillus subtilis* [63]. These findings suggest that small peptides generated by the activity of another peptidase may serve as substrates for RipB. Therefore, it is suggested that RipA and RipB display crucial differences in substrate specificity, which also support the nonredundant role of these enzymes.

Peptidoglycan hydrolyses are well‐known to be under tight regulation in order to fulfil their role in the cell cycle without becoming potentially lethal autolysins. In line with that, several post‐translational and processing steps are required for the proper endopeptidase activity of RipA. To begin with, the enzyme active‐site cleft is self‐inactivated by a prodomain that functions as a lid (Fig. 2) [62]. Experiments of cell wall degradation showed that RipA almost does not have hydrolytic activity when the lid domain is engaged on the protein [62]. This suggests that the lid domain is a blocking loop with a regulatory function and does not participate in catalysis. Based on that, RipA has been considered to be produced as a zymogen [40, 62]. On the other hand, RipB appears to retain its activity in the presence of the blocking loop, possibly due to structural differences of such prodomain region among the enzymes, allowing unobstructed access to the active site of RipB [63]. In the same report, the inhibitory role assigned to the prodomain was challenged when Böth *et al*. [63] demonstrated that full‐length RipA was also capable of hydrolysing small peptidoglycan fragments. Whether the prodomain would have to be cleaved or relocated to allow substrate‐binding and hydrolase activity was addressed when post‐translational processing of RipA was observed *in vivo*. Chao *et al*. [51] found processed RipA species within the cell wall compartment of *M. smegmatis*. These authors also confirmed that the proteolytic activation of RipA is required for division, corroborating the zymogen model. Therefore, small fragments of peptidoglycan can enter the catalytic cleft of the full‐length RipA and undergo degradation by the enzyme; nevertheless, peptidoglycanase activity for both peptidoglycan remodelling and cell division is only achieved upon RipA proteolytic activation, which means by the cleavage of its prodomain region [51]. It is important to note that the binding protein responsible for RipA activation and the exact cleavage site for blocking loop release remain unknown. Furthermore, the available three‐dimensional structure of RipA catalytic domain was obtained only as the inactive zymogenic form [62], hence conformational changes caused by the proteolytic processing still need to be addressed.

**Fig. 5:**
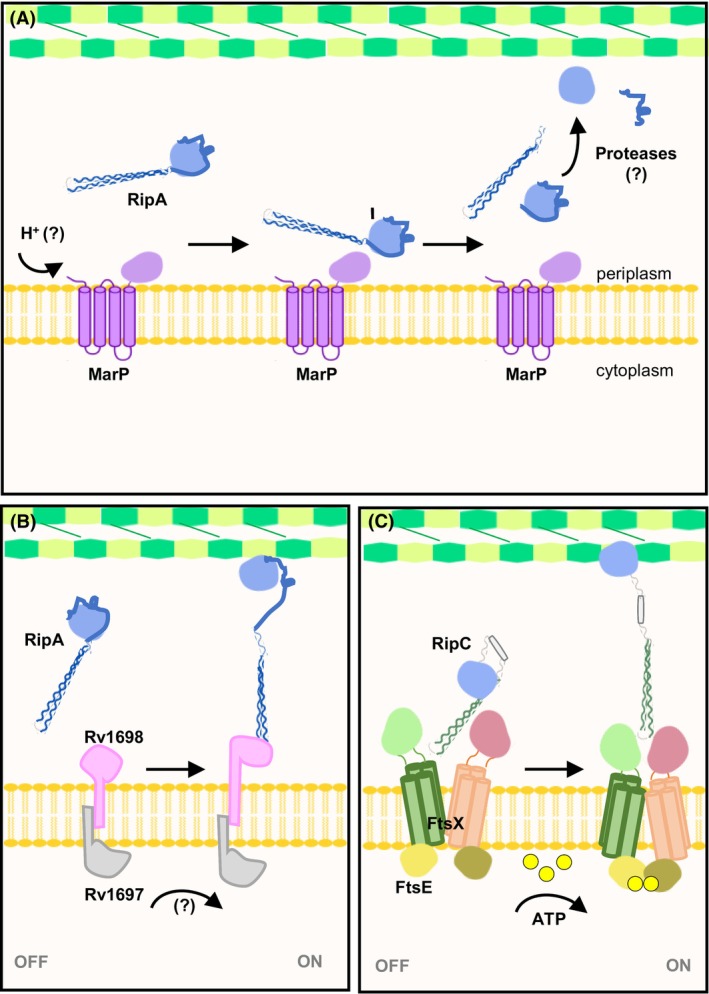
Overview of proposed mechanisms of action and regulation of RipA and RipC. (A) RipA activation based on the zymogen model. Under acidic environment, the periplasmic protease MarP (purple) is activated/senses low pH. MarP cleaves the full‐length RipA (blue), releasing the inactive catalytic domain (light blue) of RipA. The endopeptidase is further processed by unknown proteases. The cleavage of the RipA blocking loop activates its NlpC_P60 domain, which functions as a PG hydrolase. (B) RipA activation based on the FtsEX‐independent activation model for the RipA homologue Cg1735 from *C. glutamicum*. Upon a signal sensing (unknown) from the divisome, the transmembrane protein complex Rv1697:Rv1698 (grey and pink) undergoes a conformational change that releases the extracellular domain (pink) to interact with the full‐length RipA. This interaction unlocks the catalytic domain of RipA for PG hydrolysis. (C) FtsEX‐mediated activation of RipC. The FtsEX system is homologue to the Type VII ABC transporters and coordinates PG hydrolysis via signal transduction. In the presence of ATP, the cytoplasmic FtsE (purple) induces conformational changes in the transmembrane region of FtsX (orange). The extracellular domain of FtsX, in turn, binds to RipC, undergoes conformational changes and becomes activated.

The functional role of the N‐terminal domain in the RipA (absent in RipB) activity or regulation is also a matter of debate. Firstly, it was suggested the N terminus participated in the RipA activation through its proteolysis by MarP, a membrane‐associated cysteine protease, during acidic stress conditions [71] (Fig. 5A). MarP recognises the amino acid sequence AARLVAWSSE within the coiled‐coil N‐terminal domain of RipA and cleaves the peptide specifically after the Val_235_ residue [71]. However, given the work of Steiner *et al*. [64], this noncatalytic domain of RipA should be involved as a scaffold within the divisome rather than enzyme activity control since the absence of this region does not influence the catalytic site accessibility. In fact, this domain resembles a long stalk of two α‐helices (coiled‐coil structure) and seems to position the RipA catalytic region towards peptidoglycan degradation [64] (Fig. 5).

Recently, a more complex mechanism for the endopeptidase activation was proposed for the RipA homologue, Cg1735, from Cglu (Fig. 5B) [45]. Briefly, after sensing a signal from the divisome, the transmembrane complex Cg1603:Cg1604 (homologue of Rv1697/Rv1698 from *M. tuberculosis*) undergoes conformational changes that allow the extracellular core domain of Cg1604 to bind to the autoinhibited Cg1735. This interaction unlocks the catalytic domain of Cg1735 from its blocking amino‐terminal coiled‐coil domain for peptidoglycan hydrolysis. Based on that, both proteolysis and protein–protein interactions are believed to mediate RipA activation under different circumstances [45]. At neutral pH, Cg1735 would be activated by a transmembrane protein complex that senses a signal from the divisome. In contrast, proteolytic activation through MarP orthologue processing should occur in an acidic environment, such as inside phagosomes in macrophages [45, 71].

### FtsEX‐dependent NlpC_P60 enzyme—RipC

The gene *ripC* is conserved in the genomes of pathogenic species, which is in line with previous studies suggesting its participation in MTB virulence [28, 44]. Nevertheless, RipC is also encoded in the genome of several environmental mycobacteria. Thus, the role of RipC in the physiology and virulence of mycobacteria remains unclear. Likewise, there is a significant conservation of gene vicinity for *ripC* between different species of mycobacteria (Fig. 3B). For instance, *ripC* is adjacent to a gene encoding for a hypothetical protein, except in the *M. leprae* genome (Fig. 3B). Intriguingly, such protein of unknown function encoded by *rv2189c* in the MTB genome shares at least 70% sequence similarity to DUF4157 domain‐containing proteins (Table 1). According to the INTERPRO classification, proteins carrying the DUF4157 domain are putative metallopeptidases and contain the zinc‐binding HExxH motif (IPRO25295), which is conserved in all the analysed hypothetical protein sequences downstream of *ripC* (Fig. 3E). The DUF4157 domain has recently been identified as a highly conserved core domain of bacterial extracellular contractile injection system (eCIS) [72]. It can be found at the N terminus of polymorphic associated toxins or in genes adjacent to them. Although the role of DUF4157 in eCIS remains unclear, it is hypothesised that this domain would be related to the loading, release, maturation, or trafficking of toxins to the target cells [72]. Moreover, domains of novel protein effectors of the Type VI Secretion System (T6SS) seem to share similarities with DUF4157 [73, 74] in favour of the role of DUF4157‐containing proteins in toxin‐delivery systems. Nevertheless, no studies have yet explored the potential interaction between RipC and DUF4157‐containing proteins. The proposed relationship between RipC and the genes encoding DUF4157‐containing proteins is based on their genomic proximity and conservation. Therefore, whether the DUF4157 domain contributes to regulating RipC or functions as its partner in an as‐yet‐unknown pathway involved in mycobacterial physiology or pathogenesis requires further investigation.

**Table 1:** Summary of hits to Rv2189c in NCBI Blast search, using protein–protein BLAST.

Sequence ID	Description	Scientific name	Max score	Total score	Query cover	E‐value	Percent identity	Accession length
WP_240430760.1	DUF4157 domain‐containing protein [*Mycobacterium shigaense*]	*Mycobacterium shigaense*	311	311	91%	2.00E‐103	74.89	274
WP_232011294.1	DUF4157 domain‐containing protein [*Mycobacterium shigaense*]	*Mycobacterium shigaense*	310	310	91%	1.00E‐102	74.47	274
WP_139822510.1	DUF4157 domain‐containing protein [*Mycobacterium lacus*]	*Mycobacterium lacus*	308	308	88%	2.00E‐102	74.89	227
WP_232069308.1	DUF4157 domain‐containing protein [*Mycobacterium saskatchewanense*]	*Mycobacterium saskatchewanense*	309	309	91%	3.00E‐102	73.50	281
WP_250160882.1	DUF4157 domain‐containing protein [*Mycobacterium senriense*]	*Mycobacterium senriense*	305	305	89%	1.00E‐100	72.61	280
WP_231117958.1	DUF4157 domain‐containing protein [*Mycobacterium colombiense*]	*Mycobacterium colombiense*	303	303	89%	6.00E‐100	71.74	280
WP_231120855.1	DUF4157 domain‐containing protein [*Mycobacterium colombiense*]	*Mycobacterium colombiense*	303	303	89%	1.00E‐99	72.61	280
WP_231127914.1	DUF4157 domain‐containing protein [*Mycobacterium colombiense*]	*Mycobacterium colombiense*	301	301	89%	3.00E‐99	72.17	280
WP_231127247.1	DUF4157 domain‐containing protein [*Mycobacterium colombiense*]	*Mycobacterium colombiense*	301	301	89%	4.00E‐99	72.17	280
WP_231120037.1	DUF4157 domain‐containing protein [*Mycobacterium colombiense*]	*Mycobacterium colombiense*	300	300	89%	1.00E‐98	71.74	280
WP_232003550.1	DUF4157 domain‐containing protein [*Mycobacterium* sp. 1465703.0]	*Mycobacterium* sp. 1465703.0	298	298	89%	6.00E‐98	70.13	280
WP_054879829.1	DUF4157 domain‐containing protein [*Mycobacterium haemophilum*]	*Mycobacterium haemophilum*	295	295	90%	1.00E‐96	72.46	264
WP_234010113.1	DUF4157 domain‐containing protein [*Mycobacterium colombiense*]	*Mycobacterium colombiense*	295	295	89%	1.00E‐96	70.43	280
WP_248608524.1	DUF4157 domain‐containing protein [*Mycobacterium colombiense*]	*Mycobacterium colombiense*	294	294	89%	2.00E‐96	70.87	280
WP_239655127.1	DUF4157 domain‐containing protein [*Mycobacterium riyadhense*]	*Mycobacterium riyadhense*	292	292	91%	4.00E‐96	73.19	244
WP_239656711.1	DUF4157 domain‐containing protein [*Mycobacterium riyadhense*]	*Mycobacterium riyadhense*	292	292	91%	5.00E‐96	73.19	244
MCV7147333.1	DUF4157 domain‐containing protein [*Mycobacterium riyadhense*]	*Mycobacterium riyadhense*	293	293	91%	6.00E‐96	73.19	264
WP_238553432.1	DUF4157 domain‐containing protein [*Mycobacterium colombiense*]	*Mycobacterium colombiense*	291	291	89%	2.00E‐95	70.43	280
WP_047314704.1	DUF4157 domain‐containing protein [*Mycobacterium haemophilum*]	*Mycobacterium haemophilum*	290	290	89%	6.00E‐95	72.84	264
WP_186245836.1	DUF4157 domain‐containing protein [*Mycobacterium simulans*]	*Mycobacterium simulans*	285	285	92%	2.00E‐93	70.46	234
WP_067128979.1	DUF4157 domain‐containing protein [*Mycobacterium* sp. 852 002‐51971_SCH5477799‐a]	*Mycobacterium* sp. 852 002‐51971_SCH5477799‐a	285	285	89%	2.00E‐92	70.00	280
WP_062886456.1	DUF4157 domain‐containing protein [*Mycobacterium avium*]	*Mycobacterium avium*	282	282	90%	1.00E‐91	71.24	276
WP_067358257.1	DUF4157 domain‐containing protein [*Mycobacterium* sp. 1165178.9]	*Mycobacterium* sp. 1165178.9	276	276	89%	5.00E‐89	70.00	280
WP_144957311.1	DUF4157 domain‐containing protein [*Mycobacterium helveticum*]	*Mycobacterium helveticum*	258	258	90%	2.00E‐81	71.37	319

Despite acting as a peptidoglycan hydrolase, RipC seems to be enrolled in distinct cell wall biosynthesis processes compared to the other Rip enzymes. As demonstrated by Parthasarathy *et al*. [28], a *ripC* mutant strain of MTB exhibited growth defects *in vitro* and attenuation in mouse models. Interestingly, this phenotype was not associated with effects on septation, as those previously observed for *ripA* and *ripB* mutants [27, 30, 46]. In fact, both defects in vegetative growth in broth culture and decreased virulence *in vivo* were due to altered colony gross morphology and lipid composition [28]. Hence, it is suggested that RipC should be involved with the integrity and composition of the cell wall rather than in daughter‐cell separation in mycobacterial cells.

In addition, transcriptional profile studies revealed a *ripC* upregulation profile in MTB replicating in type II alveolar epithelial cells (AEC) that implicates its possible involvement in pathogen–host interaction mechanisms during primary infection [75]. This hypothesis is supported by the fact that the peptidase RipC is located in a surface‐exposed cell wall position and has been demonstrated to be immunogenic in *in vivo* studies of mouse models infected with MTB [28, 76].

Strikingly, studies revealed that RipC peptidase activity is activated through the FtsEX system, a different pathway of post‐translational regulation from those previously described for RipA [77, 78]. The FtsEX system is homologous to Type VII ABC transporters [77, 78, 79]. This system is a transmembrane component of the divisome complex, which coordinates cell division by regulating peptidoglycan hydrolysis through signal transduction [79]. Depending on the bacterial genus, this regulation occurs either via direct activation of peptidoglycan hydrolases [77, 80, 81] or through interactions with additional protein mediators [82, 83]. Briefly, the cytoplasmic FtsE initiates the system by utilising its ATPase activity, leading to conformational changes in the transmembrane partner FtsX. Subsequently, the extracellular domain of FtsX interacts with the coiled‐coil amino‐terminal region of its cognate partners [83].

In Gram‐negative bacteria, such as the model organism *E. coli*, the FtsEX complex binds to the coiled‐coil domain of the periplasmic enzyme mediator EnvC, which activates the amidases AmiA and AmiB to degrade the septal peptidoglycan [79, 82, 83]. Conversely, in mycobacteria, the endopeptidase RipC is directly activated by the FtsEX system through conformational changes upon binding [77, 78, 83]. This mechanism resembles those described for the Gram‐positive *Bacillus subtilis* (ClwO) and *Streptococcus pneumoniae* (PcsB) [80, 81].

Recent advances have provided key insights into the molecular mechanism by which RipC is activated via the FtsEX system. Cryo‐EM structures of inactive and ATP‐activated FtsEX‐RipC complexes [78] revealed the structural basis of this activation (Fig. 5C). The authors of this study proposed that RipC is recognised by FtsEX through a ‘match and fit’ process, in which the FtsX extracellular dimer displays an asymmetric response, where one domain binds to residues from the RipC ɑ1 helix and remains stationary. In contrast, the other extracellular domain rotates to fit and lock RipC ɑ2 helix underneath. As a result, RipC is positioned in an inclined binding mode that is unique compared to other FtsEX‐hydrolyses models [78]. Second, enzymatic assays showed both ATP‐binding and hydrolysis are dispensable for RipC recruitment. However, ATP hydrolysis is required for the activation of the FtsEX‐RipC system [77, 78]. In this regard, despite the lack of the lid domain as in RipA, RipC is also maintained in a self‐inhibited state. In this configuration, both the coiled‐coil domain and the linker region of RipC occludes the NlpC_P60 catalytic domain in a similar way to that previously described for Cg1735 (RipA homologue in Cglu) [45]. This active state is achieved by a series of conformational changes triggered by posthydrolysis of ATP. Curiously, the NlpC_P60 domain of RipC was not observed in the FtsEX‐RipC structure complex after activation, possibly due to the increased flexibility, which impairs direct visualisation [78].

The domain architecture of RipC resembles that of RipA, as both endopeptidases are the only Rip enzymes that carry an amino‐terminal coiled‐coil domain [28] (Fig. 2I). By contrast, the NlpC_P60 domain of RipC does not carry a regulatory prodomain. Instead, it is preceded by a proline‐rich region [28, 77, 78] (Fig. 2I). Although the signatures DCSG and GD are conserved in RipC (Fig. 4E), the enzyme carries the canonical NlpC_P60 Cys^300^‐His^348^‐His^360^ catalytic triad rather than the Cys‐His‐Glu residues found in RipA and RipB [77] (Fig. 4C).

Nevertheless, some knowledge gaps remain to be addressed. For example, are there any other partners transiently interacting with RipC in the context of the divisome complex? In addition, even though RipC shares with RipA the presence of an N‐terminal domain involved in the regulation of the catalytic domain activity, the two endopeptidases seem to be controlled under distinct post‐translational regulatory mechanisms. In line with that, RipC carries the canonical NlpC_P60 catalytic residues, differing from both RipA and RipB, in favour of the nonredundant role of such enzymes. Hence, the substrate specificity, and therefore, the physiological niche of RipC activity compared to the other Rip peptidases still needs to be investigated.

### The noncatalytic NlpC_p60 protein—RipD

Conversely, *ripD* exhibits a more divergent gene context (Fig. 3C,D). For instance, despite being conserved in the genus *Mycobacterium*, there are differences in the *ripD* neighbourhood between pathogenic and environmental species (Fig. 3C). In the genomes of most clinically relevant mycobacteria species, *ripD* is adjacent to biotin and trehalose biosynthesis gene clusters, while in *M. abscessus* and *M. smegmatis ripD* is found in other different genomic regions, such as close to *furA‐katG* loci, or *tenA* gene, respectively.

Intriguingly, *ripD* is highly immunogenic during infection. Studies detected RipD as a strong antigen in serum samples from cattle infected with *M. avium* subspecies *paratuberculosis*, the causative agent of Johne's disease in ruminants [84]. A similar result was observed in immunised mouse models treated with recombinant _MTB_RipD [85]. These findings revealed the potential of RipD as a biomarker for *Mycobacterium* infection diagnosis and vaccine development. In addition, the upregulation of *ripD* was also observed in type II AEC, analogous to that observed for *ripC* [75], which indicates that RipD should also participate in host–pathogen interaction mechanisms. Also, it was proposed that RipD could be enrolled in the dysregulation of host alternative RNA splicing [86], which is another alternative way of modulating host–pathogen interactions.

RipD is the first Rip member identified as a noncatalytic peptidoglycan‐binding protein [43, 84]. Indeed, RipD retained the ability to bind to the peptidoglycan but has evolved to an inactive amino‐terminal NlpC_P60‐like domain (Fig. 2). The loss of RipD hydrolytic activity is associated with the substitutions of the catalytic residues to the Ala^83^‐Ser^132^‐Glu^144^ triad (Fig. 4D), in which cysteine is exchanged to serine [43, 84]. Moreover, the protein carries a pentapeptide sequence repeat at its carboxy terminus, which is thought to modulate its interaction with cell wall components. Based on that, there is a hypothesis that RipD could be involved in the recruitment of other enzymes for peptidoglycan degradation [43], although this still needs experimental confirmation.

### The putative cysteine peptidase—RipE

The putative cysteine peptidase RipE (Rv0024) is the least studied enzyme in the Rip family. According to genomic comparative analysis, *ripE* (*rv0024*) gene is uniquely present in the genome of pathogenic species [44, 45]. Moreover, based on genomic context searches (Fig. 3D), *ripE* is transcribed in an operon containing three genes. The gene *rv0023* located upstream of *ripE* in the operon was recently identified as a member of the xenobiotic response element (XRE) family of transcriptional regulators and has been shown to enhance tolerance against isoniazid and ethionamide [87]. Likewise, heterologous expression of _MTB_RipE leads to isoniazid and pyrazinamide resistance in *M. smegmatis* [42]. To date, _MTB_RipE heterologous overexpression in *M. smegmatis* induced biofilm formation and resistance to anti‐TB drugs that target cell wall biosynthesis [42]. Based on that, more studies are needed in order to investigate the RipE function on the physiology and pathogenesis of mycobacteria.

RipE displays the carboxy‐terminal NlpC_P60 domain carrying the Cys‐His‐Glu catalytic triad as in RipA and RipB, as well as the motifs DCSG and GD (Fig. 4). Furthermore, at the moment, there is no structural data for RipE, highlighting the need for structural biology attempts towards this enzyme.

## NlpC_P60 endopeptidases as potential antimycobacterial drug candidate targets

Despite the importance in the biology and pathogenesis of mycobacteria, only a couple of studies targeting the mycobacterial NlpC_P60 proteins in the scope of drug discovery and development are available in the literature to date. These studies involve the *in silico* screening of small molecules against RipD [88], and natural product compounds [89] or repurposing FDA‐approved drugs against RipA [90].

Since these enzymes are involved in controlling peptidoglycan biosynthesis during cell growth and division, either the inhibition or dysregulation of their hydrolytic activity represents a promising approach for drug development. Most studies on the gene function of these enzymes highlight the effects of gene deletion, which can mimic the action of inhibitor molecules. As mentioned, the studies of Δ*ripA*, Δ*ripB* and Δ*ripC* mutants revealed significant phenotypic alterations that interfere with normal cell growth and/or division [27, 28, 30, 46]. Furthermore, the inhibition of those peptidoglycan hydrolase activities increases the vulnerability of mycobacterial cells to other antibiotics, such as the β‐lactams, underscoring the importance of these enzymes in drug repurposing studies for combating mycobacterial infections [28, 30, 46].

On the other hand, developing drug candidates that mimic protein–protein interactions and enhance the hydrolytic activity of these enzymes (such as mimicking RipA‐RpfB synergy) is also an interesting strategy. As autolysins, their hyperactivation can induce bacterial cell lysis, offering an innovative approach to fighting these infections. In addition, studies investigating the immunogenicity of the Rip enzymes represent important avenues for advancing translational research in both human and veterinary clinical contexts [91].

To the best of our knowledge, although progress has been made in information about the molecular basis of the Rip family in the control of mycobacterial cell division and pathogenesis, no wet‐lab studies on drug discovery targeting these enzymes have been published, and only a few *in silico* screening efforts have been conducted to date. Therefore, this class of proteins is still underexplored as targets for drug discovery research against mycobacterial infections.

## Conclusions

It is evident that a set of NlpC_P60‐like endopeptidases plays a key role in maintaining cellular integrity, control of daughter‐cell separation, virulence, drug tolerance, and host–pathogen interaction mechanisms in mycobacteria. To date, five NlpC_P60‐coding genes have been identified in the MTB genome and were described here as the Rip family members, which display different distribution patterns across *Mycobacterium* species. Hence, the analysis of gene and genomic context conservation can shed light on the nonredundant roles of these NlpC_P60 proteins in the physiology and pathogenesis of clinically relevant mycobacteria.

Overall, the Rip proteins exhibit differences in structure, regulation, and function. Despite the heightened interest in understanding the molecular basis of these macromolecules, information about them is still limited. RipA is the most studied NlpC_P60 enzyme from mycobacteria so far. Due to its multiple functions, RipA is considered a multifunctional protein, which implies the complexity of this endopeptidase regulation. Therefore, the understanding of these mechanisms has been quite challenging. Additionally, RipA and RipB are co‐transcribed in a highly conserved bicistronic operon and share the same catalytic domain structure. Although their inactivation loop has differences that underlie distinct substrate specificities, such extensions are unique in the protein family. Nevertheless, whether the two enzymes act in distinct pathways or are regulated in response to specific signals remains elusive. The other three Rip proteins are from clades other than RipA and RipB. The recent structure of RipC in complex with the FtsEX system clarified the mechanism of RipC activation. However, the role of RipC in the physiology and virulence of mycobacteria is not completely understood. Notably, RipC is crucial for cell wall integrity as the lack of *ripC* results in increased cell permeability and drug susceptibility. Thus, RipC has the potential to be a drug target as its inhibition may facilitate the penetration of other antimycobacterial compounds [83]. The structure of RipD revealed an amino‐terminal NlpC‐like domain modified in a noncatalytic activity. Intriguingly, RipD was shown to be the most antigenic mycobacterial NlpC‐like protein, with the potential to be explored as a biomarker for MTB serodiagnosis [91] and vaccine development against mycobacterial infections of human and veterinary clinical importance [84]. The less known Rip peptidase is RipE. Its structure has not yet been elucidated, but the enzyme seems to retain the hydrolase activity and have a role in biofilm formation and to mediate drug resistance.

## Funding statement

The Article Processing Charge for the publication of this research was funded by the Coordenação de Aperfeiçoamento de Pessoal de Nível Superior ‐ Brasil (CAPES) (ROR identifier: 00x0ma614). WOA Institution: Coordenacao de Aperfeicoamento de Pessoal de Nível SuperiorConsortia Name: CAPES 2025. [Correction added on 3rd April 2025, after first online publication: Funding Statement has been updated in this version].

## Author contributions

CSS contributed with literature review, manuscript writing and figure preparation. MVBD contributed to critical manuscript revision and final editing.
